# Causal relationship between asthma and ulcerative colitis and the mediating role of interleukin-18: a bidirectional Mendelian study and mediation analysis

**DOI:** 10.3389/fimmu.2023.1293511

**Published:** 2023-12-14

**Authors:** Xin Zou, Rui-Ling Lu, Bin Liao, Shi-Jie Liu, Shi-Xue Dai

**Affiliations:** ^1^ Department of Gastroenterology, Ganzhou Municipal Hospital(Guangdong Provincial People’s Hospital Ganzhou Hospital), Ganzhou, Jiangxi, China; ^2^ Department of Rheumatology and Immunology, The Second Clinical Medical College, Jinan University (Shenzhen People’s Hospital), Shenzhen, Guangdong, China; ^3^ Department of Gastroenterology, Geriatric Center, National Regional Medical Center, Guangdong Provincial People’s Hospital Ganzhou Hospital, Ganzhou, Jiangxi, China

**Keywords:** asthma, ulcerative colitis, interleukin-18, mediation, Mendelian randomization, tool variables, causal inference

## Abstract

**Objective:**

Numerous observational investigations have documented a correlation between asthma and ulcerative colitis(UC). In this Mendelian Randomization (MR) study, we utilized extensive summary data from Genome-Wide Association Studies (GWAS) to further estimate the association between adult-onset asthma and the risk of UC, and to investigate the role of Interleukin-18 (IL-18) as a potential mediator.

**Materials and methods:**

A two-step, two-sample MR study was conducted through mediation analysis. For this study, we employed a two-sample MR analysis using the inverse variance-weighted (IVW), weighted median, weighted mode, and MR-Egger regression techniques. We utilized publicly accessible summary statistics from a GWAS meta-analysis of adult-onset asthma in the UK Biobank (n=327,253; cases=26,582; controls=300,671) as the exposure factor. The outcomes were derived from GWAS data of individuals with European ancestry (n=26,405; cases=6,687; controls=19,718). GWAS data for IL-18 were obtained from individuals of European ancestry (n=9,785,222; cases=3,636; controls=9,781,586).

**Results:**

The MR analysis indicates that adult-onset asthma is associated with an increased risk of UC, with an odds ratio (OR) of 1.019 (95% CI 1.001–1.045, P=0.006). However, there is no strong evidence to suggest that UC significantly impacts the risk of adult-onset asthma. IL-18 may act as a potential mediator in the causal relationship between adult-onset asthma and UC, with a mediation proportion of 3.9% (95% CI, 0.6%–6.9%).

**Conclusion:**

In summary, our study established a causal relationship between asthma and UC, in which IL-18 contributes to a small extent. However, the primary factors underlying the influence of asthma on UC remain unclear. Future research should focus on identifying other potential mediators. In clinical practice, it is important to pay greater attention to intestinal lesions in patients with asthma.

## 1 Introduction

Asthma, a diverse and inflammatory respiratory condition, is closely linked to the remodeling of the airways. Individuals with asthma experience difficulty breathing and wheezing, caused by blockage and increased sensitivity of the air passages (1). Asthma is a prevalent chronic condition in childhood (2), and is also frequently found in adult populations (3). Approximately 339 million individuals worldwide suffer from asthma, with projections suggesting that this figure will rise to 400 million by 2025. Asthma is understood to be caused by a combination of different environmental factors and an individual’s genetic makeup, classifying it as a genuine multifactorial condition (4). In Europe, more than 8% of adults are afflicted with asthma. Among them, ten million individuals developed the disease before the age of 45 years (5), with the highest prevalence rates observed in countries such as the United Kingdom and Sweden (6). In the United Kingdom, one out of every twelve adults (8.3% prevalence) is affected by asthma (7).

UC is a persistent and recurring non-penetrating condition that causes inflammation in the colon, primarily impacting the large intestine. Its progression is unpredictable, typically extending continuously from the distal to the proximal end, without skip lesions. Common symptoms include sores in the mucous membranes, bloody stools, rectal spasms, and an increased vulnerability to developing colorectal cancer (8). UC shows a higher prevalence in adult males compared to females, with a ratio of around 1.5 males per female. However, in children, girls are more likely to develop UC. The peak age of onset for UC is between 30 and 40 years (9). UC affects approximately 1.5 million people in Europe and over 3 million people worldwide (10), with a prevalence rate exceeding 181.1 individuals per 100,000 in North America and Europe (11). About 24.2011% of the British population is affected by UC (12). The prevalence of this illness is increasing annually, and individuals in advanced stages are susceptible to complications, leading to a decline in quality of life. There is also a rise in mortality rates among individuals who have recently been diagnosed and those with advanced disease (13).

The pathogenesis of asthma and UC remains uncertain, despite extensive research on the genetic and environmental aspects of these conditions (14, 15). Observational studies suggest a link between long-term respiratory conditions like asthma and gastrointestinal disorders (10, 12). This phenomenon of mutual influence is termed the ‘gut-lung axis’ (16). Certain exposures during early life are associated with an increased vulnerability to respiratory illnesses and alterations in the composition of gut microbiota (17). However, the precise mechanisms responsible for the interaction between the gut and lungs remain unclear. For example, some observational studies have focused on the correlation between asthma and inflammatory bowel disease (IBD) (18). Yet, which specific disease causes the other is still uncertain. This uncertainty persists even in systematic reviews and meta-analyses. Therefore, further investigation is required to unravel the complex connections among these ailments.

To the best of our knowledge, there is currently no research on the potential pathways between asthma and UC. Previous studies have provided evidence suggesting an association between IL-18 and the pathogenesis of asthma, wherein increased IL-18 expression was found in the serum of patients (19). Additionally, another clinical study has shown that IL-18 levels are related to the severity of UC (20). Therefore, IL-18 may be a potential mediator between asthma and UC.

Over the past few years, the application of Two-Sample MR analysis has become prevalent as a robust approach for inferring causality and investigating the impact of exposure factors on various diseases (21). Mendelian Randomization utilizes genetic variations as instrumental variables (IVs) to evaluate the causal association between exposure factors and diseases, thus mitigating the influence of genetic and environmental confounders (22). This approach estimates causal effects by collecting exposure and outcome data from separate samples (23).

Through the utilization of this approach, we can enhance the precision of evaluating the influence of asthma on the susceptibility to UC and acquire additional understanding regarding the intricate correlation existing between these two medical conditions. New insights and approaches for preventing and treating UC are anticipated to be revealed by the findings of this research.

## 2 Study design

### 2.1 Choosing genetic variants and identifying data sources

The genetic variation data will be sourced from GWAS of asthma and UC, which we will utilize as datasets. The datasets usually contain numerous individual samples, genotype data, and disease-related effect sizes. We explored The IEU Open GWAS(https://gwas.mrcieu.ac.uk/), which compiles numerous summary statistics from numerous GWAS.

As the exposure factor, we chose summary data from the UK Biobank cohort for individuals with adult-onset asthma, with a sample size of 327,253 (cases=26,582, controls=300,671) (24). To conduct the Two-Sample MR analysis, we utilized genetic variants linked to asthma as IVs, obtaining summary statistics with a genome-wide significance P-value threshold of 5.00E-08.In particular, we acquired summary data for 25 single nucleotide polymorphisms (SNPs) linked to asthma. As the outcome dataset, The dataset for UC (n=26,405; cases=6,687, controls=19,718) was sourced from a European population (25).The summary statistical data for IL-18 levels were derived from published GWAS meta-analysis, which included individuals of European ancestry (n=9,785,222; cases=3,636; controls=9,781,586) (26).

### 2.2 Statistical analysis conducted for Mendelian Randomization

#### 2.2.1 Statistical analysis

In MR studies, IVW is a commonly employed method used to amalgamate causal effect estimates for each Single Nucleotide Polymorphism (SNP), assessing the impact of a specific biological factor on a particular outcome (27). Additionally, MR studies utilize MR-Egger (28) and Weighted Median methods (29) to validate and complement IVW results. MR-Egger assesses directional pleiotropy of IVs, whereas the Weighted Median method offers enhanced precision relative to MR-Egger (30). These methods, employed under varying assumptions of validity, serve to derive MR estimate and facilitate a deeper understanding of causal relationships. It is worth noting that the IVW method does not require individual-level data but can instead utilize summary data to directly compute causal effect estimates. The IVW method incorporates information from multiple genetic variants and can be regarded as a two-stage least squares or allele score analysis conducted at the individual level, which is employed as the primary approach for MR analysis in this context (31).

#### 2.2.2 Primary analysis

**Figure 1:**
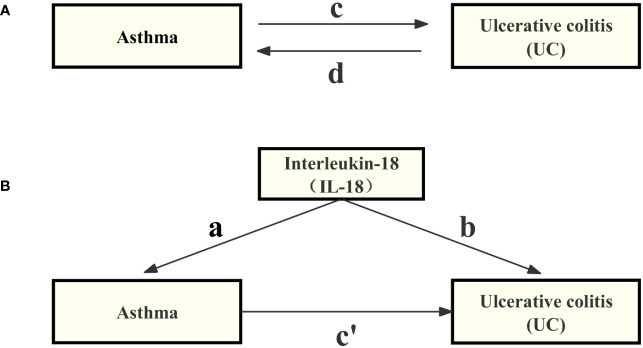
Diagrams illustrating the associations investigated in this study are provided below. **(A)** The total effect between asthma and UC, where ‘c’ represents the total effect when genetically predicted asthma is considered as the exposure, and UC as the outcome, while ‘d’ represents the total effect when genetically predicted UC is considered as the exposure, and asthma as the outcome. **(B)** The total effect is further decomposed into two components: (i) the indirect effect, which is calculated using a two-step approach (with ‘a’ denoting the total effect of asthma on IL-18, and ‘b’ representing the effect of IL-18 on UC) along with the product method (a × b), and (ii) the direct effect (c′= c – a × b). The proportion mediated is defined as the ratio of the indirect effect to the total effect.

In Mendelian randomization analysis, it is crucial that genetic variation is linked to the specific exposure and is not influenced by potential confounding factors (32). Initially, we evaluated the individual correlations between asthma and SNPs. Furthermore, we investigated the connections between each SNP and the susceptibility to UC. we utilized summary data from GWAS of asthma and UC, employing a Two-Sample MR approach (33). An overview of the study is detailed in the technical flowchart (**Figure 1**), aiming to assess the mutual causal relationship between asthma and UC (**Figure 1A**), referred to as the total effect.

Genetic IVs were constructed according to the following criteria (27, 34): (1) Genetic IVs exhibited a level of significance reaching genome-wide association threshold (P< 5.00E-08); (2) Genetic IVs demonstrated no linkage disequilibrium (LD) among them (r2< 0.001, window size = 10,000 kb); (3) Genetic IVs had a minimum minor allele frequency (MAF) > 0.01; (4) IVs located within palindromic sequences were excluded; (5) SNPs with erroneous causal directions were identified through MR Steiger filtering; (6) Genetic IVs associated with confounding factors were removed using the PhenoScanner database; (7) Outliers in the IVs were excluded using the Outlier-corrected method from the Mendelian randomization pleiotropy residual sum and outlier (MR-PRESSO) model. We excluded weak IVs characterized by an F-statistic less than 10. The formula for calculating the F-statistic is $F=R^{2}\times \frac{n-k-1}{k\times \left(1-R^{2}\right)}$ ​, where *n* represents the sample size, *k* the number of IVs used, and *R* ^2^ indicates the extent to which the IVs explain the exposure (35, 36).

#### 2.2.3 Mediation analysis

In this study, we employed a two-step MR design to conduct a mediation analysis, aiming to investigate whether IL-18 acts as a mediator in the causal pathway between asthma and ulcerative colitis (UC) (see **Figure 1B**). The total effect was decomposed into direct effects [the impact of asthma on UC without intermediaries (depicted in **Figure 1B**, path c’)] and indirect effects [the influence of asthma on UC through mediators (shown in **Figure 1B**, path a×b)]. We assessed the magnitude of the mediation effect by calculating the proportion of the indirect effect relative to the total effect. Additionally, we calculated 95% confidence intervals using the delta method to assess the reliability of our results (37). This analysis contributes to a deeper understanding of the causal relationship between asthma and UC, including the mechanisms of mediation involved.

#### 2.2.4 Heterogeneity and sensitivity test

We employed the MR Steiger filtering method to assess the causal relationships between each extracted SNP and the exposure factor, as well as the research outcomes (38). This approach calculates the variance explained in the exposure and outcomes by instrumental SNPs, verifying whether the variance in outcomes is less than that in the exposure. ‘TRUE’ MR Steiger results indicate a causal relationship in the expected direction, while ‘FALSE’ results suggest a causal relationship in the opposite direction. We excluded SNPs with ‘FALSE’ results because they significantly impacted the research outcomes rather than the exposure factor.

This study employed various methods to assess heterogeneity and horizontal pleiotropy among SNPs. We utilized Cochran’s Q statistic (39, 40), funnel plots, the MR-Egger intercept method (28), and MR-PRESSO (41) to detect and address outliers, while a random effects model was employed to evaluate the stability of results. Additionally, a leave-one-out analysis was conducted to validate the impact of each SNP on the overall causal estimates. These methodologies collectively contribute to ensuring the credibility and robustness of the MR analysis (42). All statistical analyses were conducted using R (version 4.2.3), in conjunction with the TwoSampleMR and MR-PRESSO software packages (43).

## 3 Results

### 3.1 Association of asthma with UC

In the GWAS for asthma, 25 IVs reached significance for association differences. After identifying SNPs with erroneous causal directions through MR Steiger filtering and examining LD among these IVs, one variable (rs35441874) located in a palindromic sequence was excluded. Consequently, 24 independent IVs with no LD association (r²< 0.001, window = 1000kb) were identified. After screening, we identified seven variables (rs12470864, rs17454584, rs2338821, rs6866614, rs72743461, rs7936312, and rs943689) associated with inflammatory bowel disease, and no potential confounding factors such as smoking, alcohol consumption, or body mass index were found. Using the MR-PRESSO method to remove 4 outlier values (rs2381712, rs28635831, rs4771332, rs7824278) to ensure the robustness of the assessment results. We excluded genetic variants with weak IVs (F-statistics< 10), Among them, individual SNPs explain a variance proportion of 2% to 5% for asthma. Ultimately, 13 independent SNPs were included as IVs for asthma (**Supplementary Table S1**).

**Figure 2:**
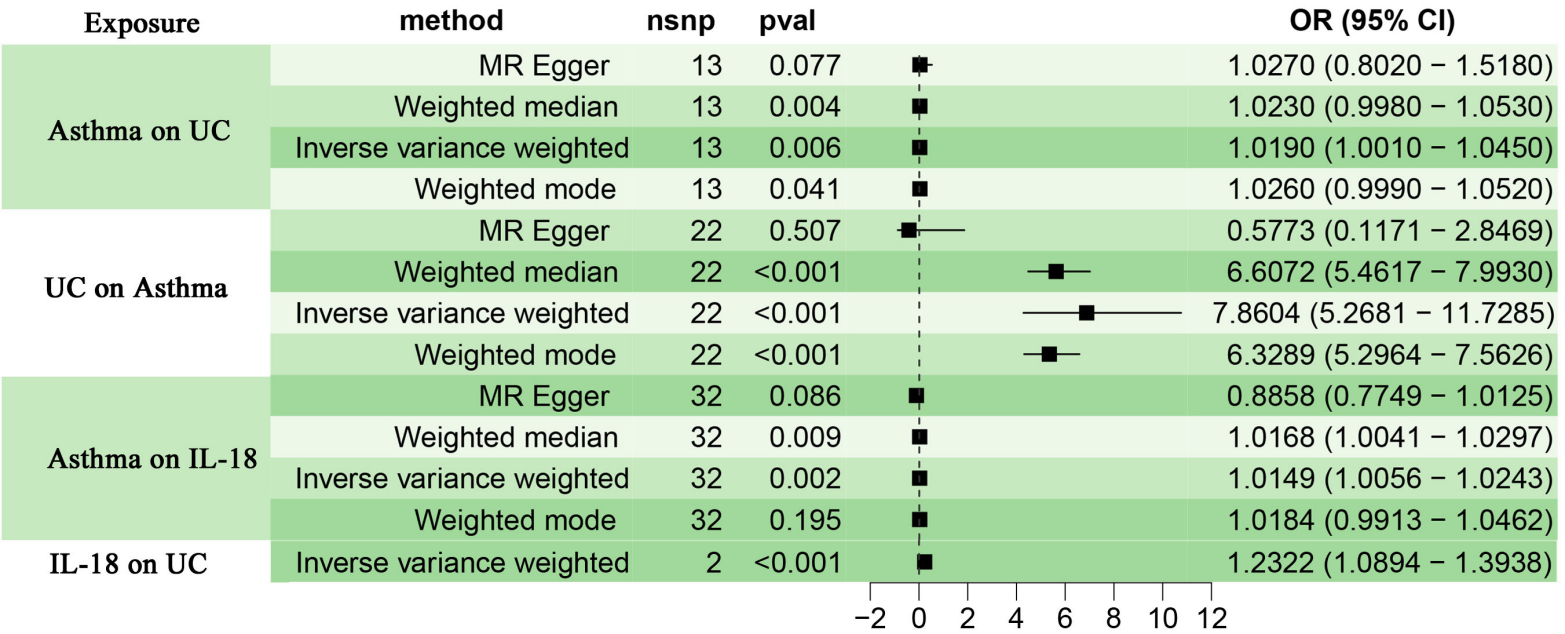
Forest plot to visualize the causal effects of IL-18 with asthma and UC.

**Figure 3:**
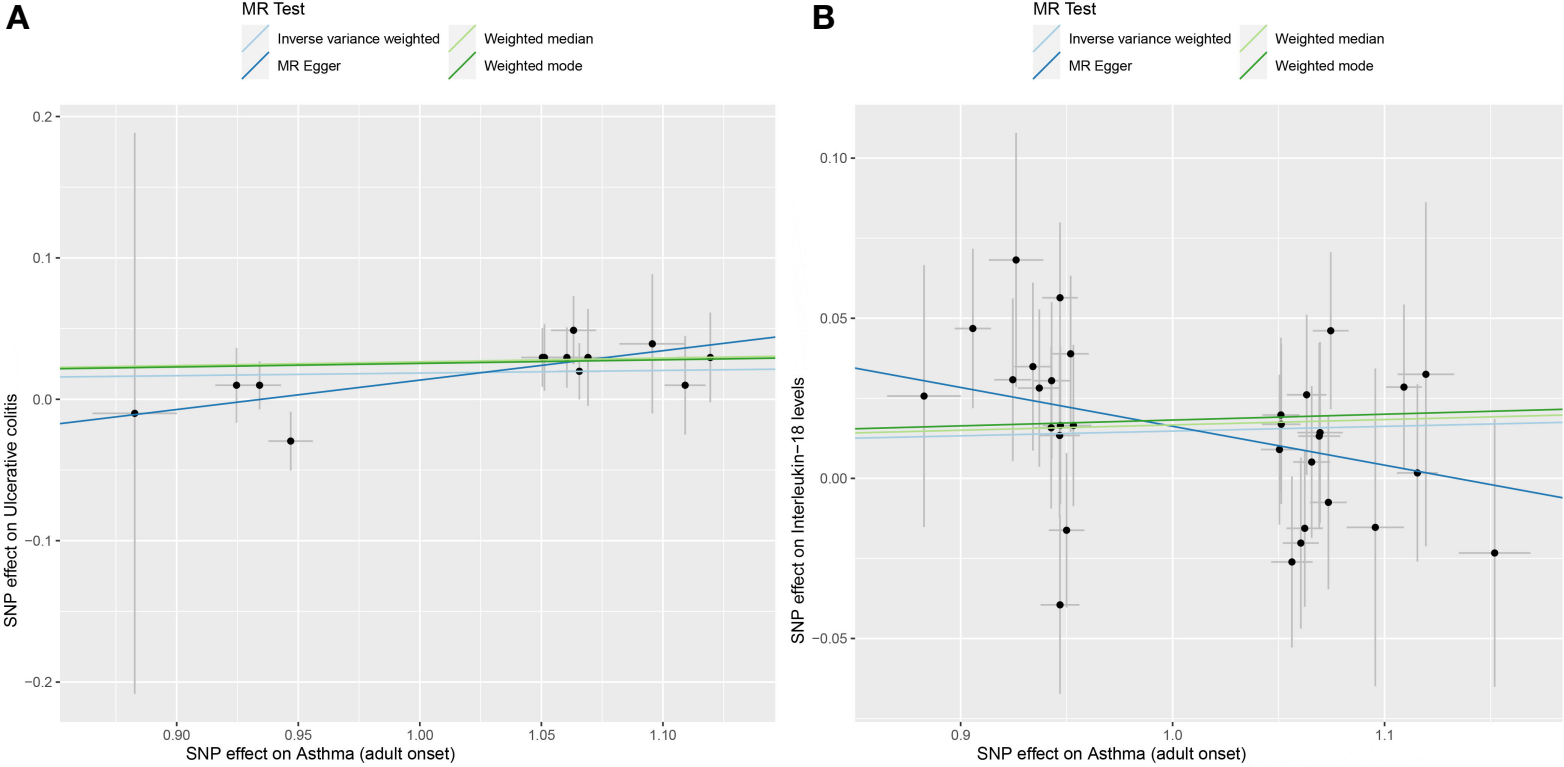
**(A)** displays scatterplot results depicting the relationship between asthma and UC in four different MR analyses, **(B)** presents scatterplot results for the relationship between asthma and IL-18 in the same four MR analyses. The slopes of each line represent the causal association for each method. The blue line represents the inverse‐variance weighted estimate, the green line represents the weighted median estimate, the dark blue line represents the Mendelian randomization‐Egger estimate, the dark green line represents the weighted mode estimate.

Choosing IVW as the primary method of analysis, the results indicate a causal relationship between asthma and UC (OR=1.019, 95%CI, 1.001-1.045, P=0.006). The weighted median method (OR=1.023, 95%CI, 0.998-1.053, P=0.004) and the weighted mode method (OR=1.026, 95%CI, 0.999-1.052, P=0.041) are consistent with the main analysis method IVW. The MR-Egger regression shows that directional pleiotropy is unlikely to bias the results (intercept=-0.194; P=0.103), but the results of the MR-Egger regression method (OR=1.027, 95%CI 0.802-1.518, P=0.077) differ from the aforementioned analysis methods. Considering that weighted median estimation has the advantage of maintaining higher estimation precision compared to MR-Egger analysis (44), the results of the MR analysis may support a potential causal relationship between asthma and UC. The forest plots and scatterplots depicting the results of the four MR methods can be found in **Figure 2** and **Figure 3**.

### 3.2 Association of asthma with IL-18

After removing palindromic and ambiguous SNPs, as well as SNPs identified through MR Steiger filtering in the incorrect causal direction, we were left with 32 genome-wide significant SNPs to use as IVs. Among them, individual SNPs explain a variance proportion of 1.4% to 5% for asthma. The F-statistic is far greater than 10 (**Supplementary Table S2**). Genetically predicted asthma was found to be positively associated with IL-18 risk according to the IVW (OR 1.0149, 95% CI 1.006-1.024,P=0.002) and weighted median methods (OR 1.02, 95% CI 1.004-1.030,P=0.009). MR-Egger (OR 0.89, 95% CI 0.775-1.013,P=0.086),weighted mode (OR 1.02, 95% CI 0.991-1.046,P=0.195). The forest plot and scatterplot of the results can be found in **Figure 2** and **Figure 3**.

### 3.3 Association of IL-18 with UC

We obtained two IVs (rs385076, rs71478720) significantly associated with IL-18 (P< 5.00E-08) from the GWAS. Among them, individual SNPs explain a variance proportion of 2.5% to 2.6% for IL-8. The F-statistic is far greater than 10 (**Supplementary Table 3**). The IVW method provided evidence of a causal relationship between IL-18 and UC (OR=1.232, 95% CI 1.089-1.393; P< 0.001)(**Figure 2**).

### 3.4 Association of UC with asthma

To assess the potential causal relationship between UC and asthma, we conducted a reverse Mendelian randomization (MR) analysis. Within the GWAS of UC, 96 IVs exhibited statistically significant differences. Addressing the issue of linkage disequilibrium among IVs, we excluded 11 IVs that were found within palindromic sequences, resulting in a set of 85 IVs free from linkage disequilibrium (r2<0.001, window=1000KB). Additionally, 39 IVs associated with confounding factors such as asthma, body weight, smoking, diabetes, and lipid levels were excluded using the PhenoScanner database. We further applied the MR-PRESSO method to eliminate 24 outlier IVs. Finally, 22 independent SNPs were included as IVs for UC. Among them, while the F-statistics are all greater than 10, the individual SNPs explain only a variance proportion of 0.1% to 0.7% for UC (**Supplementary Table 4**).

Reverse analysis revealed that the IVW (OR=7.860, 95% CI 5.268-11.729, P<0.001), weighted median (OR=6.607, 95% CI 5.462-7.993, P<0.001), and weighted mode (OR=6.329, 95% CI 5.296-7.562, P<0.001) methods all support a causal relationship between UC and asthma. However, the results of the MR Egger analysis (OR=0.577, 95% CI 0.117-2.847, P=0.507) are inconsistent with those of the three previous methods (**Figure 2**). Cochran’s Q test indicates the presence of heterogeneity (**Table 1**). The repeated application of MR-PRESSO for the detection of IVs indicated the absence of horizontal pleiotropy, as suggested by the Global Test P-value of 0.32 (**Table 1**). However, the MR-Egger regression analysis revealed the presence of horizontal pleiotropy (Intercept = 0.138; P = 0.004) (**Table 1**). Therefore, while the primary IVW analysis suggests an association between UC and asthma, the presence of horizontal pleiotropy in the IVs may lead to less robust results and potential false positives. Furthermore, the individual SNPs exhibit a relatively weak capacity to explain the genetic effects on UC (**Supplementary Table 4**). Thus, we conclude that there is insufficient genetic evidence to support a causal relationship between UC and asthma in genetic prediction.

**Table 1:** Heterogeneity and pleiotropy in MR analyses.

Exposure	Outcome	Method	SNPs	Heterogeneity	Horizontal pleiotropy			
				Q p-value	MR-Egger regression			MR-PRESSO
					Egger intercept	SE	p-value	Global test p-value
Asthma	UC	MR Egger	13	0.944	-0.194	0.109	0.103	0.43
Asthma	UC	Inverse variance weighted	13	0.793				
Asthma	IL-18	MR Egger	32	0.860	0.138	0.001	0.055	0.749
Asthma	IL-18	Inverse variance weighted	32	0.729				
IL-18	UC	MR Egger	NA	NA	NA	NA	NA	NA
IL-18	UC	Inverse variance weighted	2	0.888				
UC	Asthma	MR Egger	22	0	0.138	0.359	0.004	0.32
UC	Asthma	Inverse variance weighted	22	0				

MR, Mendelian Randomization; MR-PRESSO, MR-Pleiotropy Residual Sum and Outlier method. IL-18, Interleukin-18; UC, ulcerative colitis; NA, Not Applicable.

### 3.5 Proportion of the association between asthma and UC mediated by IL-18

**Figure 4:**
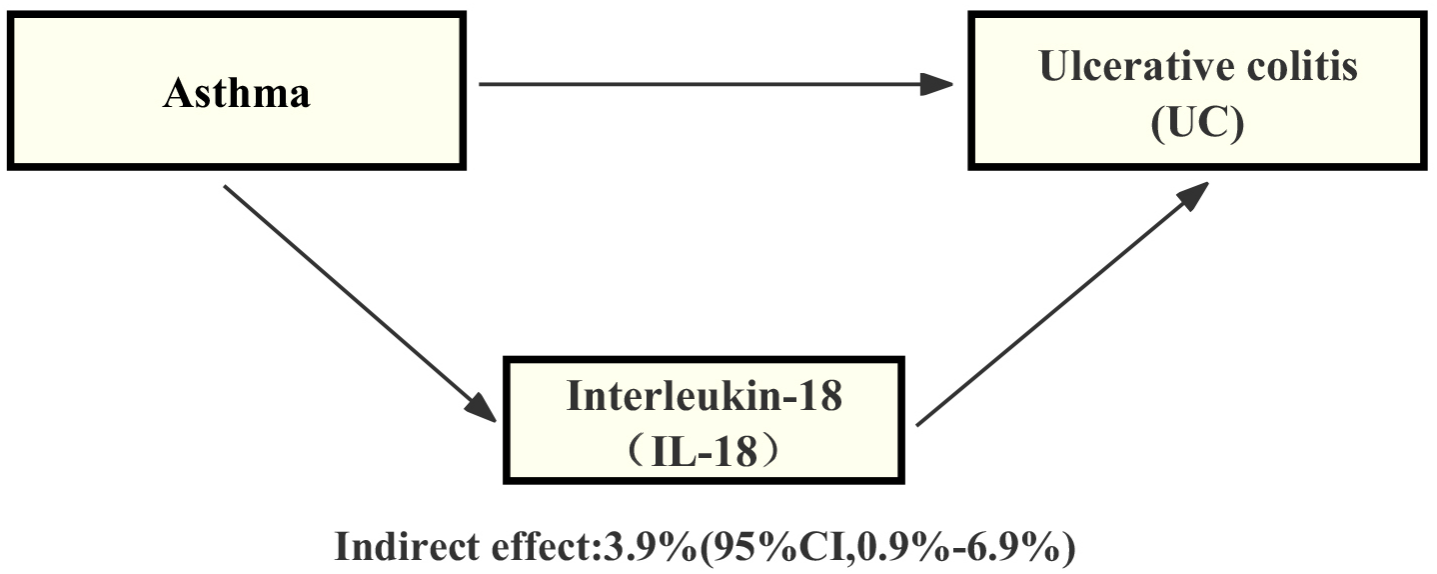
Schematic diagram of the IL-18 mediation effect.

Through our analysis, we have identified that IL-18 plays a significant role in the pathway from asthma to UC. Specifically, we have observed a certain association between asthma and an increase in IL-18 levels, and this increase in IL-18 is also linked to an elevated risk of UC. By employing mediation analysis and the delta method for computation, the results indicate that IL-18 explains 3.9% (mediation proportion: 3.9%, 95% CI, 0.9% - 6.9%) of the increased risk of UC-associated asthma (**Figure 4**).

### 3.6 Sensitivity analysis

In order to assess and correct for pleiotropy in causal estimation, we conducted sensitivity analyses in Section 3.4 of this paper concerning the causal relationship between UC and asthma. Additionally, we performed sensitivity analyses for two sets of MR studies, namely, the relationships between asthma and UC, and asthma and IL-18. Using Cochran’s Q-test and funnel plots, no evidence of heterogeneity or asymmetry among these SNPs within the causal relationships was observed (**Table 1** and **Supplementary Figure S1**). The MR-PRESSO global test did not detect potential horizontal pleiotropy (**Table 1**). Furthermore, we employed leave-one-out analysis to validate the impact of each SNP on the overall causal estimation (**Supplementary Figure S1**). After individually removing each SNP, we performed MR analysis on the remaining SNPs. The consistent results suggest that the inclusion of all SNPs significantly contributes to the establishment of causal relationships. Additionally, in the MR study of IL-18 on UC, due to the limited number of IVs (only two), MR-Egger regression analysis and MR-PRESSO global tests could not be conducted for sensitivity analysis. Therefore, we utilized the Wald ratio method to test for horizontal pleiotropy for each SNP (45). The results consistently showed no evidence of horizontal pleiotropy (rs385076 P=0.019, rs71478720 P=0.018) (**Supplementary Table 3**).

## 4 Discussion

Multiple research studies have indicated that people who suffer from asthma are more prone to developing UC compared to the overall population (18). Nevertheless, the connection between asthma and UC remains unclear. In order to tackle this inquiry, we performed an analysis of MR utilizing four distinct estimation techniques: IVW, weighted median, Weighted mode, and MR-Egger regression. The findings of our study suggest that there is a cause-and-effect connection between asthma and UC (OR=1.019, 95% CI 1.001–1.045, p=0.006).Despite the variation in the MR estimates acquired from IVW, MR-Egger, Weighted mode and weighted median analyses, both IVW, Weighted mode and weighted median analyses provide evidence for a causal link between asthma and UC. Especially when taking into account the greater accuracy in estimating with the weighted median estimator in comparison to MR-Egger analysis (29). The findings of our study provide additional evidence for the potential link between asthma and the likelihood of developing UC. Hence, our discoveries validate the previously noted association between asthma and UC in observational investigations. In addition, our research results indicate that IL-18 plays a mediating role in this causal relationship, accounting for approximately 3.9%. However, in reverse MR studies, due to the presence of horizontal pleiotropy in IVs and insufficient explanatory power for genetic effects, there is insufficient evidence to establish a causal relationship between UC and asthma.

In a retrospective cohort study based on population, the occurrence of UC and Crohn’s disease was assessed in individuals who had asthma. The findings of this research suggest that asthma could have an influence on the progression of UC, with potential involvement of various mechanisms (18). The potential cause of the disease is the breakdown of immune tolerance and consequent immune damage, resulting in heightened sensitivity to environmental triggers frequently seen in intestinal and respiratory conditions (46). The gut and respiratory tract originate from the same embryonic structure and have similarities in terms of epithelium, glands, and lymphoid tissue (47). Bronchoalveolar lavage and bronchial biopsies frequently reveal signs of subclinical pulmonary inflammation in individuals diagnosed with UC and Crohn’s disease (48, 49).To summarize, both the intestinal and respiratory epithelial cells originate from the same embryonic source, and it is believed that the correlation between asthma and UC is caused by the disturbance from possible immune and environmental factors (46, 47).Nevertheless, as not all individuals with asthma experience UC, there might still be some uncertainty regarding the cause-and-effect connection between asthma and UC. The study conducted by L-X Chen et al. (50) revealed that, in an analysis of 200 asthma patients, the examination of IL-18 levels showed significantly higher results compared to the control group. These findings indicated a notable association between the expression levels of serum IL-18 and its genetic polymorphism with asthma. In another study (20), it was demonstrated that the average plasma concentration of IL-18 in patients with active UC (422 ± 88pg/mL) was twice that of the healthy control group (206 ± 32pg/mL). This result suggested a close correlation between the activity of UC and the concentration of interleukin-18 in plasma, which aligns with the outcomes of our own research.

The study’s robustness is derived from the utilization of Mendelian randomization, which mitigates the inherent biases that may exist in observational studies (51). Nevertheless, Even when employing the Mendelian randomization method in research, the problem of pleiotropy (correlation) bias remains unresolved (52). The presence of genetic variations can be linked to various phenotypes, referred to as ‘pleiotropy,’ which has the potential to complicate and influence causal estimates (53). Including multiple variations in MR analysis can enhance statistical power, but it may also introduce invalid IVs and pleiotropy (54). Hence, it is necessary to employ sensitivity analysis techniques in order to verify the accuracy of the findings obtained from MR studies. To tackle pleiotropy, we employed weighted median estimation, which yields accurate estimates even when half of the SNPs are not valid instruments (44).Furthermore, MR-Egger regression was employed to examine the presence of unequal pleiotropy and assess the influence of exposure on outcomes. Despite the potential decrease in accuracy and effectiveness, the outcomes of our weighted median estimation align closely with the IVW estimation results, thus enhancing our confidence in these connections. The data we have corroborate prior observational research indicating a link between asthma and UC. The present research results offer possible ways to assess the influence of asthma on the likelihood of developing UC. Simultaneously, a two-step two-sample MR study conducted through mediation analysis revealed that IL-18 is a potential mediator in the causal relationship between asthma and UC.

In recent years, research has confirmed the significant role of IL-18 in the pathogenesis of asthma (19). IL-18, a cytokine closely related to the IL-1 family, plays a crucial role in fine-tuning cellular immunity. It serves as both an auxiliary factor in the development of Th2 cells and the production of IgE, and it is vital in the differentiation of Th1 cells. Studies have found increased expression of IL-18 in the serum of asthma patients, with an association between IL-18 polymorphism and susceptibility to asthma, highlighting its potential importance in asthma treatment. Additionally, the role of IL-18 in UC has gained new understanding. A study investigating the mechanistic role of IL-18 in colitis induced by dextran sulfate sodium (DSS) used a DSS-induced colitis mouse model to examine its functional role (55). This study found that IL-18’s pro-inflammatory effects became more pronounced in the later stages of the disease. From these studies, we observe that IL-18’s role differs between asthma and UC: in asthma, elevated levels of IL-18 are associated with disease activity, while in UC, IL-18 exerts pro-inflammatory effects. These findings suggest that monitoring and regulating IL-18 levels may be crucial in the management of both diseases. For asthma patients, controlling the disease and monitoring IL-18 levels can contribute to disease prevention and early detection and may also reduce the risk of developing UC. This is particularly significant given that UC, a challenging-to-treat disease, can lead to severe complications, such as an increased risk of tumors in later stages. Therefore, these studies offer new insights into the combined management of asthma and UC.

There are several limitations to this study. Firstly, the impact of genetic variations on the mentioned exposure factor is limited, as they can only account for a small portion of the variability in a specific exposure (56). Therefore, our analysis may have limited ability to detect associations. Secondly, the investigation concerning asthma,IL-18 and UC relies on individuals of European descent. It is crucial to conduct additional MR studies on diverse populations to account for potential racial disparities and selection biases that could impact causal relationships. Thirdly, this research utilized a pair of GWAS summary repositories and did not possess data at the individual level, thereby making it unfeasible to conduct subgroup analysis based on age or gender and compare variations in causal impacts among subgroups. Fourthly, We acknowledge that due to sample size limitations, the instrumental variables we utilized may not have been sufficient to provide higher statistical power, particularly within smaller subgroups (57). Additionally, despite our efforts to address potential pleiotropy and minimize horizontal pleiotropy, the complete elimination of such effects in MR analysis remains challenging.

## Author’s note

All authors fulfill the requirements of the Editorial Board of the International Committee of Medical Journal Editors (ICMJE) and are accountable for the credibility of the complete work, having given their approval.

## Data availability statement

The datasets presented in this study can be found in online repositories. The names of the repository/repositories and accession number(s) can be found below: https://gwas.mrcieu.ac.uk, ebi-a-GCST007799 https://gwas.mrcieu.ac.uk, ebi-a-GCST000964, and https://gwas.mrcieu.ac.uk,ebi-a-GCST004441.

## Author contributions

XZ: Writing – original draft, Writing – review & editing. RL: Writing – review & editing. BL: Writing – review & editing. SL: Formal Analysis, Writing – review & editing. SD: Writing – review & editing.

## Supplementary material

Supplementary Figure 1 Figures C and C’ respectively display funnel plots and leave-one-out plots for the asthma and UC MR analyses, while Figures D and D’ respectively present funnel plots and leave-one-out plots for the asthma and IL-18 MR analyses. The blue line represents the inverse-variance weighted estimate, while the dark blue line represents the MR-Egger estimate.
