# Supplementary figures and images for: Causal relationship between asthma and ulcerative colitis and the mediating role of interleukin-18: a bidirectional Mendelian study and mediation analysis
Source: Front Immunol. 2023 Dec 14;14:1293511. doi: 10.3389/fimmu.2023.1293511 (PMC10757619; doi:10.3389/fimmu.2023.1293511)

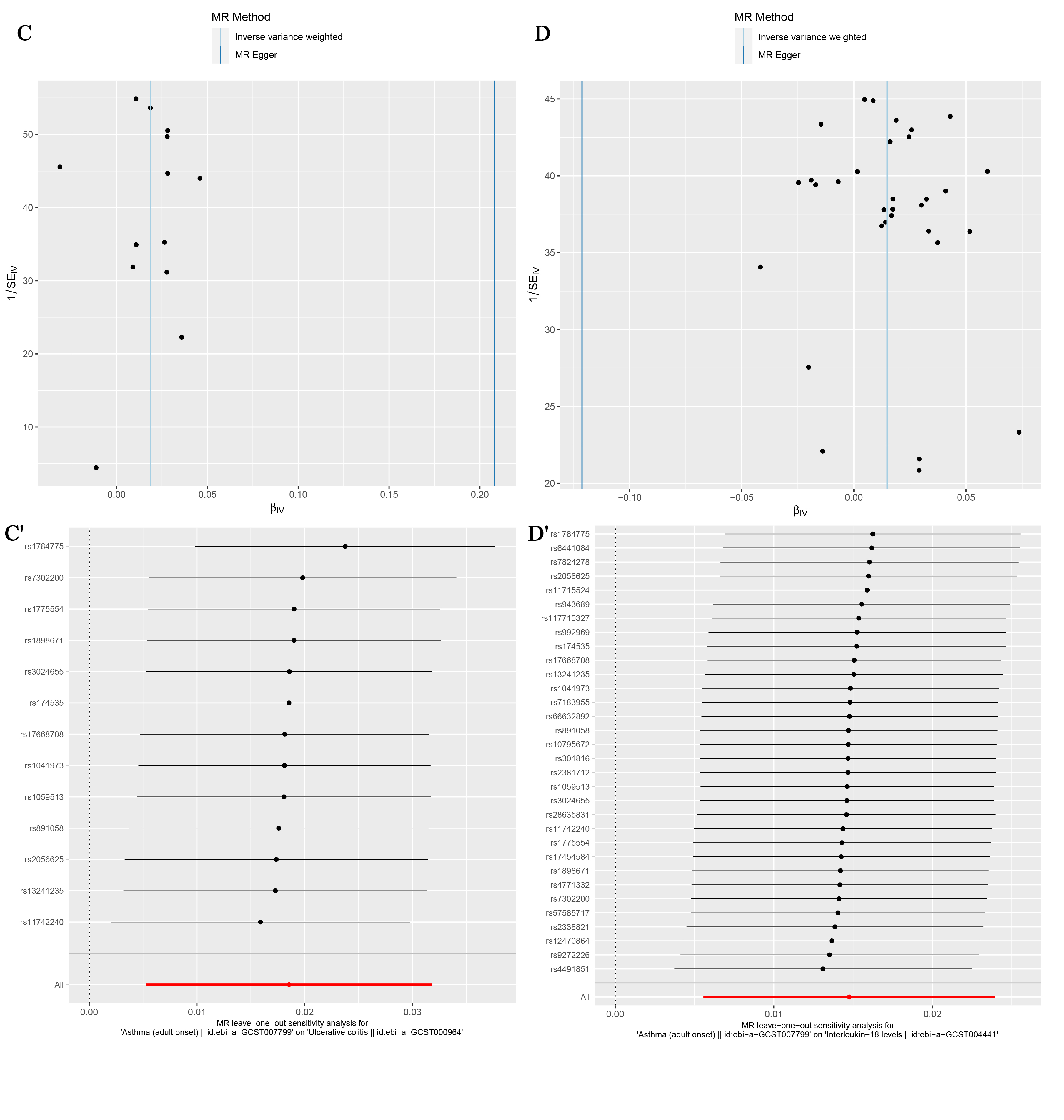

Supplement: Supplementary Figure 1 — Figures C and C’ respectively display funnel plots and leave-one-out plots for the asthma and UC MR analyses, while Figures D and D’ respectively present funnel plots and leave-one-out plots for the asthma and IL-18 MR analyses. The blue line represents the inverse-variance weighted estimate, while the dark blue line represents the MR-Egger estimate. [file Image_1.jpeg]
